# Device color influences e-cigarette flavor expectations, perception, and appeal

**DOI:** 10.1093/chemse/bjag007

**Published:** 2026-02-27

**Authors:** Ina M Hellmich, Reinskje Talhout, Sanne Boesveldt

**Affiliations:** Centre for Health Protection, National Institute for Public Health and the Environment (RIVM), Bilthoven, The Netherlands; Division of Human Nutrition and Health, Wageningen University, Wageningen, The Netherlands; Centre for Health Protection, National Institute for Public Health and the Environment (RIVM), Bilthoven, The Netherlands; Division of Human Nutrition and Health, Wageningen University, Wageningen, The Netherlands

**Keywords:** crossmodal perception, sensory integration, color–flavor association, retronasal olfaction, electronic cigarettes, flavor appeal

## Abstract

Color influences flavor expectations and experiences in foods. For example, red can enhance perceived sweetness and green can enhance sourness. Whether color similarly affects flavor perception in e-cigarettes remains unknown. As flavor strongly contributes to e-cigarette appeal, understanding the role of color may inform product regulation. We used a 3 (device color: red, green, reference) × 2 (flavor: tobacco-flavored, flavorless) × 3 (route of administration: seeing, smelling, vaping) mixed design to investigate the influence of e-cigarette color on flavor perception in 63 e-cigarette users. Brown and white devices served as reference colors. Participants rated hedonic and analytical flavor attributes on 101-unit visual analog scales. In the seeing condition, the flavor of red and green e-cigarettes was expected to be liked (Δ_Max_ = 22) and wanted (Δ_Max_ = 20) more than the flavor of a reference device [Δ_Max_ indicates the largest observed difference], and expected to be more familiar (Δ_Max_ = 23), sweet (Δ_Max_ = 28), sour (Δ_Max_ = 40), and fruity. These expectations were confirmed during use (smelling and vaping; where color effects were independent of e-cigarette flavor or route) compared to reference, red and green devices were rated higher in liking (Δ_Max_ = 5), wanting (Δ_Max_ = 6), familiarity (Δ_Max_ = 6), sourness (Δ_Max_ = 9), and fruitiness (Δ_Max_ = 18). Only the red device was rated as sweeter (Δ = 7). Red and green e-cigarettes differed only in expected sourness (seeing condition, Δ = 20). We conclude that device color influences expected and experienced e-cigarette flavor perception and appeal. These findings support regulating color as part of broader flavor restriction policies.

## 1. Introduction

Flavor perception is fundamentally multisensory (Calvert et al. 2004). Rather than operating in isolation, sensory systems interact continuously, with input from one modality shaping how information from another is interpreted (Calvert et al. 2004). How sensory input modulates flavor perception depends on past experiences and learned associations. For example, pairing a taste stimulus with an olfactory stimulus (e.g. sucrose with a strawberry odor) can influence expected and experienced sweetness of the taste stimulus, if an individual learned to associate these stimuli (Stevenson et al. 1995; Stevenson and Boakes 2003; Wang et al. 2024). Integrating sensory input with learned information stored in memory helps humans not only to react to their immediate environment but also to anticipate the future. These crossmodal correspondences are well documented and have implications for product design and consumer behavior (Garber et al. 2001; Spence 2020).

Not only olfaction, also vision—particularly color— has been shown to influence flavor perception. In food and beverage contexts, color can shape both flavor expectations (Wei et al. 2012; Velasco et al. 2016; Woods and Spence 2016), and sensory experiences, including perceived sweetness (Clydesdale et al. 1992; Bayarri et al. 2001; Calvo et al. 2001), sourness (Pangborn and Hansen 1963; Fernández-Vázquez et al. 2014), and overall flavor intensity (Chan and Kane-Martinelli 1997; Zampini et al. 2008). These effects extend beyond the product itself: packaging color alone can bias flavor expectations, and, in turn, flavor experiences. In fact, color may be one of the most influential visual cues in shaping flavor expectations when applied to packaging (Piqueras-Fiszman and Spence 2015).

How color influences flavor perception depends not only on the specific color and personal learning histories (Levitan et al. 2008; Shankar et al. 2010) shaped by culture (Levitan et al. 2014) but also on the broader context of product presentation and product category. For example, red packaging may evoke expectations of sweetness in yoghurt drinks while in the context of meat products, the same color may suggest fattiness (Tijssen et al. 2017). These color-induced expectations play a key role in shaping the flavor experience, but their effects are moderated by additional factors. One such factor is the degree of congruency between the expected and the actual sensory input. When expectations are in line with experience, assimilation occurs—perception is biased toward the expectation. That is, a sweet-tasting red-colored beverage may be experienced as sweeter than it is, if the red color induced a sweetness expectation. In contrast, when expectations strongly diverge from actual input, contrast effects occur, biasing perception away from expectations (Yeomans et al. 2008). For instance, if a red-colored beverage tastes bitter, the mismatch between the sweet expectation and the bitter experience may lead to an even lower sweetness rating than if no color cue had been given at all. Contextual factors such as the sensory modalities through which flavor information is delivered may also play a role (Koza et al. 2005).

Notably, the route of administration (ROA)—and thus whether a product is being perceived as internal (e.g. in the mouth) or external (outside the body)—modulates the multisensorial context and may influence how color affects the flavor experience. A key distinction in odor perception is whether an odor is experienced orthonasally, via the nostrils during sniffing, or retronasally, when the odor travels from inside the mouth to the olfactory epithelium during ingestion. Prior studies suggest that color may enhance odor perception when a beverage is smelled orthonasally (i.e. perceived as external), but suppress it when the same stimulus is experienced retronasally (i.e. perceived as internal) (Koza et al. 2005). Whether this reflects a contrast effect, possibly driven by the heightened salience of internal stimuli, remains unclear. It is well established, however, that the same odor can evoke qualitatively different percepts depending on whether it is delivered orthonasally or retronasally (Rozin 1982; Pellegrino et al. 2021).

Whether color–flavor interactions extend beyond food contexts remains largely unknown. Most products that elicit a flavor percept—defined as the integrated sensation arising from taste, retronasal olfaction, tactile, and thermal cues—are food-related. In feeding contexts, it is evolutionarily adaptive for humans to use color cues to predict a food's nutritional value and prepare the body for efficient digestion (Ott et al. 2012; Wang et al. 2024). Whether similar predictive mechanisms are involved for non-food products that also evoke flavor sensations is unclear. One study on odor perception found that color influenced odor intensity ratings of liquid body soap (Gatti et al. 2014), suggesting that crossmodal associations extend beyond food. However, it is still poorly understood how color influences flavor perception in other non-food items that—unlike body soap—are brought into the body and may engage multiple sensory modalities, including retronasal olfaction. These may include oral care products, inhalers, pacifiers, and condoms.

E-cigarettes provide an interesting but understudied case in crossmodal sensory research. These devices vaporize e-liquids that typically contain a range of flavorings (Krüsemann et al. 2021a). The resulting aerosol is inhaled and stimulates flavor perception—primarily via retronasal olfaction but also through gustatory stimulation (Rosbrook et al. 2017), chemesthesis (Talavera et al. 2009; Johnson et al. 2022), and possibly through tactile and thermal cues. As e-cigarette use poses health risks (Glantz et al. 2024), and flavors increase their appeal (Audrain-McGovern et al. 2016; Villanti et al. 2017), many jurisdictions have implemented flavor bans to limit e-cigarette uptake, particularly among youth (Reiter et al. 2024). Likewise, e-cigarette color stimulates product interest and use (Kistler et al. 2019; Okawa et al. 2020; Smith et al. 2023; Gomes et al. 2024). Youth, for example, show greater willingness to try branded and colorful e-cigarettes, or e-cigarettes presented in branded and colorful packaging, compared to plain designs (e.g. black [Gomes et al. 2024], white [Tattan-Birch et al. 2025], or olive [Simonavičius et al. 2024]). Yet, e-cigarette color remains unregulated, despite its potential to enhance product appeal through visual aesthetics (Kistler et al. 2019; Okawa et al. 2020; Smith et al. 2023; Gomes et al. 2024). Whether device color can enhance product appeal also through enhances in flavor appeal is not studied yet.

This study aimed to assess whether and how the color of e-cigarette devices influences expected and experienced flavor perception and appeal. As many e-cigarettes are fruit-flavored (Havermans et al. 2021), and based on established color–flavor associations, we hypothesized that, compared to a white or brown reference device, participants would (i) expect the flavor of the red device to be sweeter (Johnson et al. 1983), consistent with associations between red colors and berry flavors (Zampini et al. 2007); (ii) expect the flavor of the green device to be more sour, consistent with green colors and lime associations (Zampini et al. 2007); (iii) expect the flavor of both the red and green devices to be more fruity; and finally, (iv) expect the flavor of both the red and green devices to be liked more, as fruity flavors are typically rated as more appealing (Krüsemann et al. 2021b). We further hypothesized that smelling a flavorless e-liquid would produce flavor experiences that are in line with and biased toward these expectations (assimilation), and that smelling a tobacco-flavored e-liquid may produce flavor percepts that deviate from expectations and therefore produce flavor experiences that are biased away from them (contrast). Finally, drawing on findings from the beverage domain, where the influence of color differs between orthonasal and retronasal exposure, we hypothesized that the effect of color on flavor perception may differ between smelling and vaping.

## 2. Material and methods

### 2.1 Participants

Participants were recruited in Wageningen and surroundings through posters, flyers, e-mails, and the university website. Participants were included if they gave their informed consent, were between 18 and 55 yr old, in good general health, proficient in English and therefore able to understand the study material, with normal sense of smell (tested during screening; see section: screening session) and if they vaped at least once a month. Participants were excluded from participation if they reported allergies to compounds used in the study, if they were breastfeeding and/or (planning to become) pregnant. Participants were excluded from analysis if their score for the Ishihara color blindness test indicated moderate or strong red-green deficiency (tested during debriefing) (Ishihara 1985). A sample size of *n* = 60 was targeted to find a medium sized effect (dz = 0.5; reflects a 10-unit difference on a 101-unit VAS-scale, assuming a 20-unit SD of differences) with a probability of 80% (determined a priori using GPower 3.1, selecting a 2-tailed paired-samples *t*-test and a Bonferroni-adjusted α level of 0.0042 (α = 0.05/12 comparisons). We included 63 participants to account for potential data exclusion due to color-blindness. The study was approved by the Research Ethics Committee of WUR (Approval number: 2024-132-A) and conducted in accordance with the Declaration of Helsinki (World Medical Association 2013).

### 2.2 Design

This study used a 3 (e-cigarette color: red, green, reference [brown and white]) × 2 (flavor: tobacco-flavored, flavorless) × 3 (route of administration: seeing, smelling, vaping) mixed design to investigate the influence of color on flavor perception in e-cigarettes. Brown (tobacco-flavored) and white (flavorless) e-cigarettes served as reference colors.

### 2.3 Products

**Figure 1:**
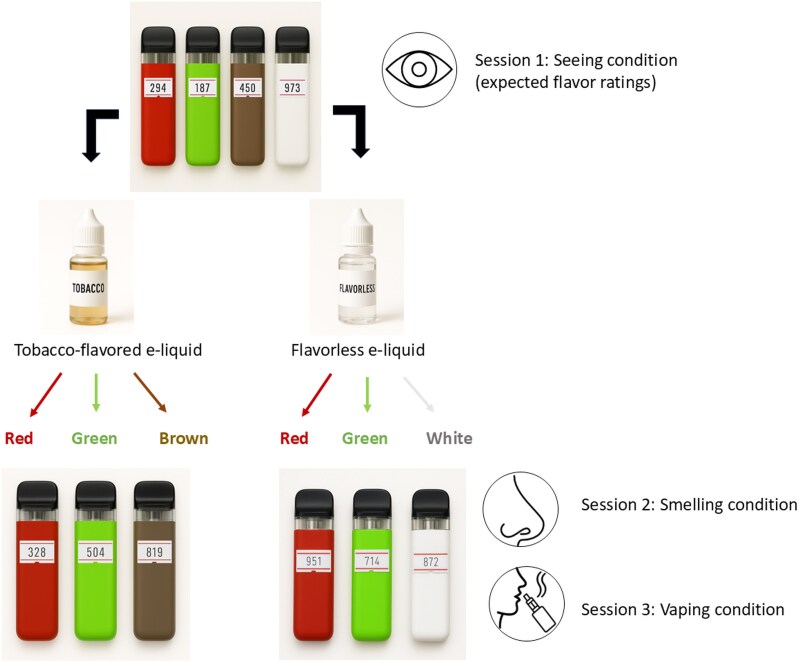
Schematic presentation of the device color, flavor, and route of administration combinations used in the study.

We used commercially available SMOK novo 2X refillable e-cigarettes (800 mAh; approximately 45 g) that we spraypainted red, green, brown, or white. For the smelling and vaping sessions (see Study Procedure—Test sessions), the e-cigarette pods (SMOK novo 2x meshed 0.8 Ω MTL) were filled with 1 mL of either a tobacco-flavored e-liquid (Dutch Blend, Liqua) or a flavorless base e-liquid (Bookwill Base), both nicotine-free. Post-hoc chemical analysis detected no flavorings in the flavorless e-liquid and 2 flavorings in the tobacco-flavored e-liquid, 2-hydroxy- 3,5,5-trimethyl- 2-cyclohexenone, and (E)-beta-damascone (for more information of the chemical analysis, see Supplementary Materials). Red and green e-cigarettes were tested with both tobacco-flavored and flavorless e-liquids. Brown e-cigarettes were exclusively paired with tobacco-flavored e-liquid, and white e-cigarettes with flavorless e-liquid, to reduce the number of trials and minimize participant fatigue. A clean mouthpiece was used for each participant to ensure hygiene. Fig. 1 shows an overview of the 6 e-cigarette conditions used.

### 2.4 Study procedure

#### 2.4.1 Screening session

After giving informed consent, completing the screening questionnaire, and meeting the eligibility criteria, participants performed the Sniffin’ Sticks 16-item Odor Identification test (Burghart, Holm, Germany). This test requires participants to select a descriptor for 16 common odors from 4 options each (Rumeau et al. 2016). In line with earlier work(Morquecho-Campos et al. 2021; Yang et al. 2023), a passing score was set at ≥11 correct responses—slightly below the normative cut-off of 12 (Oleszkiewicz et al. 2019) to account for potential unfamiliarity with certain odors in the Sniffin’ Sticks test battery among international participants (e.g. turpentine; Catana et al. 2012; Oleszkiewicz et al. 2016; Demir et al. 2021).

#### 2.4.2 Test sessions

Participants attended 3 test sessions, separated by at least one day. In session one, they visually inspected 4 unfilled e-cigarettes—one for each color (red, green, brown, white)—and rated their expected flavors (seeing condition). In sessions 2 and 3, they evaluated 6 filled e-cigarettes: 3 filled with tobacco-flavored e-liquid and 3 filled with flavorless e-liquid, one for each color condition (red, green, reference). In session 2, participants smelled the mouthpiece of each device (smelling condition); in session 3, they took 2 puffs from each e-cigarette in the same way they normally vape (vaping condition). In all sessions, e-cigarettes were presented sequentially, one at a time, and in a randomized order across participants. Participants were not informed about the e-cigarette flavors at any point during the study. E-cigarette flavors were rated on (expected or perceived) sweetness, sourness, bitterness, fruitiness, harshness, intensity, liking, wanting, and familiarity, using 101-unit visual analog scales. These scales were continuous, unnumbered, and horizontal lines, anchored only by verbal descriptors at each end (“not at all” at the left and “very much” at the right). The scales were presented in a fixed order for each condition using the software Eyequestion (version 6.0.5; Logic8 BV). Participants indicated their response by clicking a mark anywhere along the line. To minimize sensory interference, participants were instructed to abstain from alcohol on test days and from hallucinogenic substances on the test day and the day before (Kometer and Vollenweider 2018). On smelling and vaping days, they were further asked to avoid scented body products, and to refrain from eating, drinking (except water), chewing gum, brushing their teeth, smoking, or vaping for 1 h before testing. Adherence to these instructions was confirmed via a questionnaire at the start of each session.

#### 2.4.3 Debriefing

After the vaping session, participants were asked to guess the true aim of the study in an open-ended question. They were then informed about the actual aim and completed the Ishihara color blindness test (Ishihara 1985). A final questionnaire addressed their habitual e-cigarette use, cigarette smoking history, and the cultural background most influential to their sensory perception. Participants were informed that the latter typically refers to the culture in which someone grows up. Awareness of study aim, e-cigarette use frequency, smoking frequency, and cultural background were tested as covariates in the statistical analysis.

## 3. Data analysis

All analyses were conducted in IBM SPSS Version: 28.0.1.1. We computed a binary variable, *awareness of study aim*, indicating whether participants correctly identified the study's true purpose. Open-ended responses mentioning terms such as color, appearance, or visual design (e.g. “*how the e-cigarette design influences preference*”) were coded as correct.

### 3.1 Expected flavor rated in seeing condition

To test whether expected flavor ratings differed by color, we tested several mixed models with participant as a random effect and e-cigarette color (red, green, white, brown) as a fixed effect. A full model initially included covariates (typical e-cigarette device used, e-cigarette use frequency, age, gender, cultural background, smoking frequency and awareness of study aim), which were removed stepwise if non-significant. None remained significant, so the final models retained only participant and color. Post-hoc pairwise comparisons were corrected for multiple testing using Bonferroni. Assumptions for linear modeling were evaluated; no violations were observed.

### 3.2 Experienced flavor rated during smelling and vaping

To test whether experienced flavor ratings differed by color, we fitted several full-factorial mixed models. Each model included participant as a random effect and e-cigarette color (red, green, reference), flavor (tobacco, flavorless), and ROA (smelling, vaping) as fixed effects. In these models, brown and white devices were combined into a single reference category to enable a fully factorial analysis. We initially included the full set of main effects and interaction terms, and removed nonsignificant interactions terms and main effects one by one. Across all models, no significant 3-way or 2-way interactions involving color were found, indicating that color effects on flavor perception were consistent across flavors and ROA. As our primary interest was whether the effect of color varied by flavor or ROA, we retained significant main or interaction effects involving flavor and ROA in the models to improve fit but do not interpret these effects further. Expected means for these conditions are reported in the Supplementary Table S1. We then included covariates (same as for expected flavor models, described above), which were removed stepwise if non-significant. To address violations of normality of the error terms, we applied a square root transformation to 4 of the dependent variables (sourness, bitterness, harshness, and familiarity). Means, but not standard errors were back-transformed to the original scale (by squaring) for ease of interpretation. Post-hoc pairwise comparisons were corrected for multiple testing using Bonferroni.

## 4. Results

### 4.1 Participants

Participants had a mean age of 23 ± 4 yr (SD) and 49% identified as female, 48% as male, and 3% as other. The majority (73%) identified European culture as the most influential in shaping their crossmodal associations. Additional participant information is displayed in Table 1.

**Table 1:** Participant characteristics (*n* = 63).

Participant characteristic		Frequency (%)
Age (mean ± SD)	23.4 ± 3.8 yr	
Gender		
	Male	30 (47.6)
	Female	31 (49.2)
	Other	2 (3.2%)
Awareness of study aim	Yes	33 (53.4%)
	No	30 (47.6%)
Cultural background		
	European	46 (73.0)
	Asian	15 (23.8)
	Other	2 (3.2)
E-cigarette use frequency		
	Daily	16 (25.4)
	Multiple times a week	21 (33.3)
	Once a week	22 (34.9)
	At least once a month	4 (6.3)
Typical e-cigarette device use		
	Disposable	33 (52.4)
	Rechargable and refillable	19 (30.2)
	Rechargable, non-refillable	10 (15.9)
	I don’t know	1 (1.6)
Cigarette smoking frequency		
	More than once a week	19 (30.2)
	Once a month—once a week	20 (31.7)
	Less than once a month	24 (38.1)

### 4.2 Expected flavor rated during seeing condition

**Figure 2:**
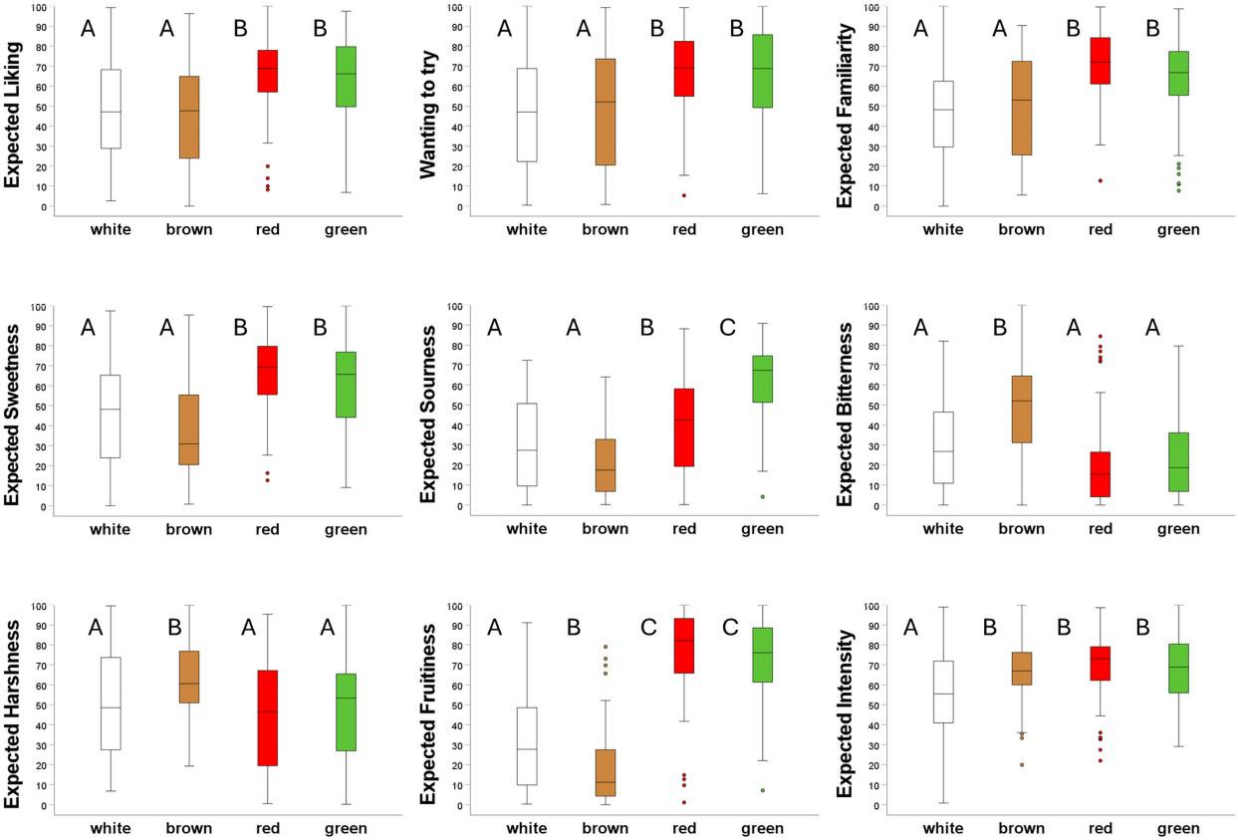
Boxplots showing raw flavor expectation ratings after visual inspection for each of the 4 e-cigarette colors (seeing condition). The e-cigarette device colors are shown on the x-axes. The horizontal line within each box indicates the median rating. Boxes represent the interquartile range (IQR), spanning the 25th to the 75th percentile of the data. Whiskers extend to the most extreme values within 1.5 × IQR. Values beyond the whiskers (if any) are shown as individual data points. Dissimilar letters indicate differences in means (*P* < 0.05).

Participants rated the flavor of red and green e-cigarettes higher on expected liking compared to the brown e-cigarettes (Δ_MAX_ = 22.3 ± 4.0; *P* < 0.001) and white e-cigarettes (Δ_MAX_ = 18.2 ± 4.0; *P* < 0.001; where Δ_Max_ indicates the largest mean difference between either red or green and a reference color). They were also more willing to try the red and the green e-cigarettes compared to the reference colors (Δ_Max_ = 19.5 ± 4.1; *P* < 0.001). Participants expected the flavor of the red and green e-cigarette to be more familiar (Δ_Max_ = 22.5 ± 3.5; *P* < 0.001), sweeter (Δ_Max_ = 28.4 ± 4.2; *P* < 0.001), more fruity (Δ_Max_ = 57.5 ± 3.7; *P* < 0.001), and more sour (Δ_Max_ = 40.4 ± 3.5; *P* < 0.001) compared to reference. Red and green e-cigarettes differed from the brown in all attributes except expected flavor intensity, and from the white in all but expected bitterness and harshness. They differed from each other only in expected sourness (Δ = 20.7 ± 3.5; *P* < 0.001). Compared to the brown e-cigarette, the white e-cigarette was expected to be more fruity, less intense, less bitter, and less harsh. Supplementary Table S2 shows model information such as estimated means and standard errors per flavor attribute and e-cigarette color condition. Fig. 2 visualizes the distribution of raw (unmodeled) flavor expectation ratings for each of the 4 e-cigarette colors.

### 4.3 Experienced flavor rated during smelling and vaping

**Figure 3:**
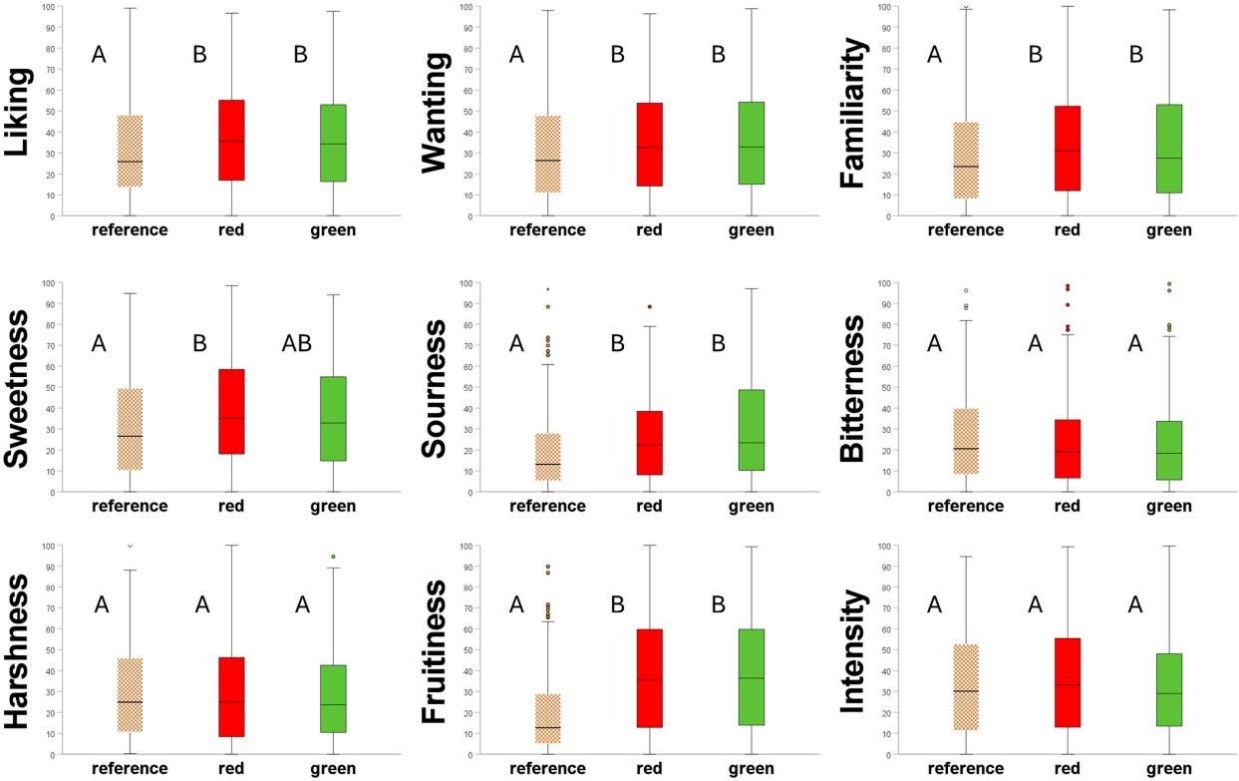
Boxplots showing raw flavor experience ratings for each of the 3 e-cigarette color conditions. The e-cigarette colors are shown on the x-axes. The horizontal line within each box indicates the median rating. Boxes represent the interquartile range (IQR), spanning the 25th to the 75th percentile of the data. Whiskers extend to the most extreme values within 1.5 × IQR. Values beyond the whiskers (if any) are shown as individual data points. Brown and white e-cigarettes served as the reference. Dissimilar letters indicate differences in means (*P* < 0.05).

Across all models, no significant interactions involving color were found, indicating that color effects on flavor experience were consistent across flavors (tobacco-flavor and unflavored) and ROA (smelling and vaping). For both flavor conditions and ROA, the flavors of red and green e-cigarettes were liked more (Δ_Max_ = 5.2 ± 1.7; *P* = 0.009), wanted more (Δ_Max_ = 6.5 ± 1.8; *P* = 0.001), more familiar (Δ_Max_ = 6.2 ± 0.2; *P* = 0.003), more sour (Δ_Max_ = 9.1 ± 0.2; *P* < 0.001), and more fruity (Δ_Max_ = 18.1 ± 1.9; *P* < 0.001). Only the red e-cigarette was rated as sweeter compared to the reference (Δ = 7.2 ± 1.8; *P* < 0.001). Flavor ratings between green and red e-cigarettes did not differ. All e-cigarettes were rated equally bitter, harsh, and intense. Supplementary Table S3 shows model information such as estimated means and standard errors per flavor attribute and e-cigarette color condition. Fig. 3 visualizes the distribution of raw (unmodeled) flavor experience ratings for each of the 3 e-cigarette color conditions.

## 5. Discussion

We conducted a sensory evaluation of differentially colored e-cigarettes in *n* = 63 regular users to assess how device color influences expected and experienced flavor perception and liking. As hypothesized, the flavor of red and green e-cigarettes was expected to be liked more, and indeed liked more, compared to the flavor of white and brown devices. Similar effects were observed for wanting and familiarity. In line with common color–flavor associations, and in line with our hypothesis, the red-colored e-cigarette was expected to be and experienced as being sweeter compared to the reference colors (white and brown), while the green-colored e-cigarette was expected to be and experienced as being more sour. Both e-cigarettes were also expected to be and experienced as being more fruity, again confirming our hypothesis. Moreover, red and green e-cigarettes were *expected* to be less harsh and less bitter compared to the brown e-cigarette, and more intense compared to the white e-cigarette. Red and green e-cigarettes were, however, not *experienced* as less harsh, less bitter and more intense compared to the reference. Taken together, our findings support the idea that color can shape both flavor expectations and flavor experiences in the context of inhaled products.

We found no evidence that the influence of color on flavor perception differed between flavorless and tobacco-flavored e-cigarettes. Evidence from research in the food domain shows that the impact of sensory expectations on sensory perception depends on the similarity between the expected and actual sensory input. Perception often aligns with expectations when they are consistent with the sensory input (assimilation). In cases of clear discrepancy, however, perception may be biased in the opposite direction (contrast) (Yeomans et al. 2008). Based on this framework, one might hypothesize that a red-colored e-cigarette—evoking sweetness expectations—could enhance sweetness perception when paired with a flavorless e-liquid, which consists of a mixture of propylene glycol and glycerin, with a mildly sweet taste (Rao et al. 2018; Leffingwell and Associates) (assimilation). In contrast, when paired with a tobacco-flavored e-liquid that does not match the sweetness expectation, the same color cue might suppress perceived sweetness through a contrast effect. Our findings did not support this pattern, suggesting either that expectation–experience discrepancies were insufficient to elicit contrast effects, or that this framework does not extend from food to e-cigarettes. Similarly, but unlike previous studies on colored beverages (Koza et al. 2005), we found no evidence that the influence of color on flavor perception differed between orthonasal and retronasal routes of administration (smelling and vaping). This suggests that device color may influence not only users’ flavor perception but, in a similar manner and to a comparable extent, also the perceptions of bystanders exposed to the sight and smell of e-cigarettes. As such, color may enhance e-cigarette flavor appeal even among non-users, whose protection remains a central goal of e-cigarette flavor regulations.

## 6. Policy implications

Although the observed effects of color on *experienced* flavor appeal were modest, from a regulatory standpoint they are meaningful, especially given the large influence on flavor *expectations*. In jurisdictions such as the Netherlands, explicit flavor descriptors on e-cigarette packaging are prohibited (NVWA, accessed 2025). However, our findings show that color alone can serve as a potent flavor cue, shaping both what consumers expect and how they ultimately perceive the product. In consequence, device color may circumvent the regulatory aim of flavor bans and standardizing color may reduce e-cigarette appeal. This is especially true regarding that, in addition to serving as a flavor cue, color can also increase e-cigarette appeal through design aesthetics (Kistler et al. 2019; Okawa et al. 2020; Smith et al. 2023; Gomes et al. 2024; Simonavičius et al. 2024; Tattan-Birch et al. 2025). Standardizing device color could therefore reduce both sensory-driven and design-based appeal, strengthening the case for color regulation as part of broader tobacco control and flavor restriction policies.

## 7. Strength and limitations

A key strength of this study is the integration of expectation and experience ratings, which allowed us to assess how color shaped both anticipated and actual flavor experiences. Since expectations can drive initial e-cigarette use—and nicotine addiction may develop rapidly in adolescents, even after limited exposure—reducing anticipated appeal is a critical regulatory goal. Additionally, even though not tested statistically, our design supports the interpretation that experienced flavor perception and liking were, at least in part, driven by color-induced visual expectations. However, including a session of visual examination may have inadvertently increased participants' awareness of the study aim. Unlike the smelling and vaping sessions—where attention may have been drawn to the sensory properties of the e-cigarettes—visual inspection relied solely on the device's appearance, with color being the only distinguishing feature. Indeed, about half of participants correctly guessed the study's purpose. Yet, awareness of study aim was not significant as covariate in any of the models, indicating that this awareness did not influence the observed color effects. This is consistent with findings by Zampini et al., who demonstrated that color cues can influence flavor perception even when participants are explicitly warned that such cues may be misleading (Zampini et al. 2007). A limitation of the study design is the pooled reference condition (brown and white) to model flavor experiences, which may have obscured differential effects on flavor attributes like bitterness, intensity, and harshness. While red and green vapes were *expected* to be less harsh and less bitter than the brown device specifically, combining brown and white devices into a single reference category for *experienced* flavor ratings potentially diluted these effects.

## 8. Recommendations for further research

Future studies may benefit from employing a fully crossed design, where brown and white e-cigarettes are paired with both flavors (tobacco and flavorless), to allow the analysis of their individual color contributions. Additionally, testing a broader variety of e-liquid flavors could help clarify whether the congruency between flavor expectations and actual sensory input—and thus, mechanisms of assimilation and contrast—modulates the effect of color on flavor perception in e-cigarettes. It would also be valuable to explore the combined effects of visual and verbal cues. In food research, color and flavor labels have been shown to exert additive effects on flavor perception (Shankar et al. 2009). For example, brown color vs. green color and a label of “dark chocolate” vs. “milk chocolate” both independently enhanced ratings of *chocolatey-ness* in a sensory analysis of chocolate candies (M&Ms). Similar mechanisms may operate in e-cigarette products. Manipulating both device color and label information (e.g. naming the flavor “Berry Mix” or “Classic Tobacco”) could clarify how multisensory and cognitive cues interact in shaping e-cigarette flavor perception and appeal.

Furthermore, future studies could investigate the mechanisms by which device color affects flavor appeal. In particular, it remains to be tested whether color-induced changes in analytical attributes (e.g. in fruitiness) mediate the observed effects of color on flavor liking and wanting. Although our design allowed for such an analysis, and pilot runs indicated that the influence of color on flavor liking is fully mediated by colors influence on fruitiness, sweetness, and familiarity, time constraints prevented us from conducting a statistically sound mediation analysis. We encourage future research to examine these pathways, which could clarify whether color increases flavor appeal via changes in specific sensory attributes. Such knowledge may improve the targeting of regulatory measures aimed at reducing the appeal of nicotine products.

Finally, given that tobacco control policies aim primarily to prevent uptake among non-users, future studies should assess how non-users perceive e-cigarette flavor cues. Importantly, non-users may interpret color cues differently than regular users. For example, while a brown-colored device may evoke tobacco associations among experienced users, non-users might associate the same color with chocolate—leading to distinct flavor expectations and potentially greater appeal. Investigating user-group differences in color–flavor associations could help anticipate the broader impact of standardizing device color and inform more inclusive regulatory strategies. While it would be unethical to ask non-users to vape, our findings suggest that expectation ratings can serve as informative proxies for flavor perception during use.

## 9. Conclusion

We showed that device color shapes both flavor expectations and flavor experiences, influencing how users like and want e-cigarette flavors. Red- and green-colored devices were associated with enhanced sweetness, sourness, fruitiness, and overall appeal, indicating that visual design elements can serve as potent flavor cues. These findings highlight the need to consider color in e-cigarette flavor regulation. Specifically, standardizing e-cigarette devices to white or brown colors may reduce their flavor appeal. Importantly, studies should also examine how color–flavor associations vary across user groups, including non-users, whose protection is a central goal of tobacco control policies.

## Supplementary Material

bjag007_Supplementary_Data

## Data Availability

Data are available on request.
