# Supplementary material for: Device color influences e-cigarette flavor expectations, perception, and appeal
Source: Chem Senses. 2026 Feb 27;51:bjag007. doi: 10.1093/chemse/bjag007 (PMC13016966; doi:10.1093/chemse/bjag007)
Supplement: bjag007_Supplementary_Data [file bjag007_supplementary_data.docx]

## Supplementary Materials

### Chemical Analysis of e-liquids

We diluted the e-liquid samples 1:20 (v/v) in acetonitrile containing benzene D_6_ as an internal standard prior to GC-MS analysis. Samples were analyzed using an Agilent Technologies single quadrupole GC-MS system (7890B GC coupled with a 5977C MS), featuring an electron ionization (EI) source set at 230 °C and a quadrupole mass analyzer maintained at 150 °C. Mass spectra were acquired in full scan mode across a mass range of 30–250 m/z at a scan rate of 3.3 scans per second. The system operated under constant flow conditions at 1.0 ml/min, with an inlet temperature of 200 °C. Samples were injected in 1 µl volumes using split mode at a 10:1 ratio.

Chromatographic separation was performed on an Inertcap Aquatic-2 capillary column (0.25 mm × 60 m × 1.40 µm). The gas chromatograph oven temperature was initially maintained at 50 °C for 6 minutes, then increased at a rate of 10 °C per minute to 250 °C, followed by a 9-minute hold at this final temperature. The total runtime was 35 minutes.

No additives or flavorings prohibited under Dutch legislation were detected [43]. Both e-liquids were confirmed to be free of nicotine. The tobacco-flavored e-liquid contained two flavoring compounds, both present below a signal-to-noise ratio of 5.5 and compliant with the Dutch list of 16 permitted flavorings:

- 2-Hydroxy- 3,5,5-trimethyl- 2-cyclohexenone (CAS number 4883-60-7)
- (E)-beta- Damascone (CAS number 23726-91-2).

## Results

|  | **Tobacco** | **Flavorless** | **Smelling** | **Vaping** |
| --- | --- | --- | --- | --- |
| Liking | 37.3 ± 2.0 | 32.9 ± 2.0 | 36.8 ± 2.0 | 33.4 ± 2.0 |
| Wanting | 37.0 ± 2.1 | 32.1 ± 2.1 | 37.4 ± 2.1 | 31.7 ± 2.1 |
| Familiarity* | 30.3 ± 0.2 | 22.2 ± 0.2 | n.s. | n.s. |
| Sourness* | 21.8 ± 0.2 | 17.9 ± 0.2 | 21.7 ± 0.2 | 17.9 ± 0.2 |
| Bitterness* | 22.7 ± 0.2 | 16.3 ± 0.2 | n.s. | n.s. |
| Harshness* | 27.9 ± 0.2 | 19.7 ± 0.2 | 25.5 ± 0.2 | 21.0 ± 0.2 |
|  | **Tobacco - Smelling** | **Flavorless - Vaping** | **Flavorless - Smelling** | **Tobacco - Vaping** |
| Sweetness | 39.2 ± 2.2 | 34.0 ± 2.2 | 31.6 ± 2.2 | 35.5 ± 2.2 |
| Fruitiness | 39.0 ± 2.1 | 29.3 ± 2.1 | 29.2 ± 2.1 | 29.7 ± 2.1 |
| Intensity | 44.6 ± 2.0 | 29.1 ± 2.0 | 24 ± 2.0 | 39.1 ± 2.0 |

Table S1: Estimated means of experienced flavor ratings for tobacco flavored and flavorless e-cigarettes during orthonasal and retronasal exposure flavor (smelling and vaping condition). n.s. = non-significant differences between smelling and vaping

^*^ Dependent variables were square-root transformed to address violations of the normality of error terms. Means, but not standard errors are back-transformed to the original scale for ease of interpretation.

*Table S2: Expected flavor ratings (shown as mean ± SE) for colored e-cigarettes during visual inspection (seeing condition). Significant F-tests for color effects (p < 0.05) are shown in* ***bold****. None of the covariates was significant. As the final models included the effects of only participant and color, no other model information is given.*

|  | **White** | **Brown** | **Red** | **Green** | **F-test for effect of color** |
| --- | --- | --- | --- | --- | --- |
| Liking | 48.6 ± 2.9^A^ | 44.4 ± 2.9^A^ | 66.7 ± 2.9^B^ | 62.7 ± 2.9^B^ | **F(3,186) = 14.91; p < 0.001** |
| Wanting to try | 47.7 ± 3.3^A^ | 48.3 ± 3.3^A^ | 67.2 ± 3.3^B^ | 64.1 ± 3.3^B^ | **F(3,186) = 12.5; p < 0.001** |
| Familiarity | 47.8 ± 2.9^A^ | 49.2 ± 2.9^A^ | 70.3 ± 2.9^B^ | 62.7 ± 2.9^B^ | **F(3,186) = 18.93; p < 0.001** |
| Sweetness | 45.5 ± 3.0^A^ | 38.5 ± 3.0^A^ | 66.8 ± 3.0^B^ | 59.8 ± 3.0^B^ | **F(3,186) = 19.51; p < 0.001** |
| Sourness | 30.2 ± 2.6^A^ | 21.0 ± 2.6^A^ | 40.7 ± 2.6^B^ | 61.4 ± 2.6^C^ | **F(3,186) = 49.89; p < 0.001** |
| Bitterness | 29.4 ± 2.8^A^ | 49.3 ± 2.8^B^ | 21.7 ± 2.8^A^ | 24.6 ± 2.8^A^ | **F(3,186) = 23.39; p < 0.001** |
| Harshness | 50.0 ± 3.2^A^ | 62.8 ± 3.2^B^ | 45.6 ± 3.2^A^ | 48.6 ± 3.2^A^ | **F(3,186) = 6.89; p < 0.001** |
| Fruitiness | 30.9 ± 2.7^A^ | 18.4 ± 2.7^B^ | 75.8 ± 2.7^C^ | 72.4 ± 2.7^C^ | **F(3,186) = 123.27; p < 0.001** |
| Intensity | 54.8 ± 2.3^A^ | 66.9 ± 2.3^B^ | 69.2 ± 2.3^B^ | 68.0 ± 2.3^B^ | **F(3,186) = 10.01; p < 0.001** |

*Table S3: Experienced flavor ratings (shown as mean ± SE) for colored e-cigarettes during orthonasal and retronasal exposure (smelling and vaping condition). Significant F-tests for color effects (p < 0.05) are shown in* ***bold****.*

|  | **Reference** | **Red** | **Green** | **F-test for effect of color** |
| --- | --- | --- | --- | --- |
| Liking^1^ | 31.7 ± 2.1^A^ | 36.9 ± 2.1^B^ | 36.7 ± 2.1^B^ | **F(2,697) = 5.70;** **p < 0.003** |
| Wanting^2^ | 30.9 ± 2.3^A^ | 35.4 ± 2.3^B^ | 37.3 ± 2.3^B^ | **F(2,697) = 7.08; p < 0.001** |
| Familiarity*^3^ | 22.3 ± 0.2^A^ | 28.5 ± 0.2^B^ | 27.7 ± 0.2^B^ | **F (2,690) = 6.38; p = 0.002** |
| Sweetness^4^ | 31.4 ± 2.1^A^ | 38.6 ± 2.1^B^ | 35.2 ± 2.1^AB^ | **F(2,698) = 7.96; p <0.001** |
| Sourness*^5^ | 15.0 ± 0.2^A^ | 20.8 ± 0.2^B^ | 24.1 ± 0.2^B^ | **F(2,697) = 19.93; p < 0.001** |
| Bitterness*^6^ | 21.3 ± 0.2^A^ | 18.7 ± 0.2^A^ | 18.3 ± 0.2^A^ | F(2,690) = 2.19; p = 0.112 |
| Harshness*^7^ | 24.6 ± 0.19^A^ | 23.4 ± 0.2^A^ | 22.5 ± 0.2^A^ | F(2,688) = 0.82; p = 0.439 |
| Fruitiness^8^ | 20.0 ± 2.0^A^ | 37.4 ± 2.0^B^ | 38.0 ± 2.0^B^ | **F(2,698) = 59.20; p < 0.001** |
| Intensity^9^ | 34.5 ± 1.9^A^ | 35.7 ± 1.9^A^ | 32.9 ± 1.9^A^ | F(2,688) = 1.30; p = 0.273 |

^*^ Dependent variables were square-root transformed. Means, but not standard errors are back-transformed to the original scale for ease of interpretation.

^1^ model included Intercept [F(1,64) = 355.97; p < 0.001], Color [F(2,697) = 5.70; p = 0.003], Flavor [F(1,697) = 9.67; p = 0.002] and ROA [F(1,697) = 5.67; p = 0.018]

^2^ model included Intercept [F(1,64) = 313.36; p < 0.001], Color [F(2,697) = 7.08; p < 0.001], Flavor [F(1,697) = 11.37; p < 0.001], and ROA [F(1,697) = 15.80; p < 0.001].

^3^ model included Intercept [F(1,62) = 952.40; p < 0.001], Color [F(2,690) = 6.38; p = 0.002], and Flavor [F(1,690) = 27.63; p < 0.001]

^4^ model included Intercept [F(1,64) = 373.99; p < 0.001], Color [F(2,698) = 7.96; p <0.001], Flavor [F(1,698) = 9.35; p = 0.002], ROA [F(1,698) = 0.17; p = 0.68], and the Flavor × ROA interaction [F(1,698) = 4.33; p = 0.038]

^5^ model included Intercept [F(1,64) = 720.80; p < 0.001], Color [F(2,697) = 19.93; p < 0.001], Flavor [F(1,697) = 10.49; p = 0.001], and ROA [F(1,697) = 9.25; p = 0.002].

^6^ model included Intercept [F(1,61) = 362.64; p < 0.001], Flavor [F(1,690) = 26.64; p < 0.001], Color [F(2,690) = 2.19; p = 0.112], and Frequency of Use [F(1,61) = 7.91; p = 0.007].

^7^ model included Intercept [F(1,61) = 357.83; p < 0.001], Flavor [F(1,688) = 36.41; p < 0.001], ROA [F(1,688) = 16.64; p < 0.001], Flavor × ROA interaction [F(1,688) = 9.79; p = 0.002], Color [F(2,688) = 0.82; p = 0.439], and Frequency of Use [F(1,61) = 5.35; p = 0.024]

^8^ model included Intercept [F(1,64) = 356.47; p < 0.001], Color [F(2,698) = 59.20; p < 0.001], Flavor [F(1,698) = 10.95; p < 0.001], ROA [F(1,698) = 8.97; p = 0.003], and the Flavor × ROA interaction [F(1,698) = 9.20; p = 0.003].

^9^ model included Intercept [F(1,60) = 26.01; p < 0.001], Flavor [F(1,688) = 105.98; p < 0.001], ROA [F(1,688) = 0.13; p = 0.723], Color [F(2,688) = 1.30; p = 0.273], Flavor × ROA interaction [F(1,688) = 11.45; p < 0.001], Frequency of Use [F(1,60) = 5.17; p = 0.027], and Gender [F(1,60) = 11.72; p = 0.001].
